# *Aedes aegypti* resistance development to commonly used insecticides in Jakarta, Indonesia

**DOI:** 10.1371/journal.pone.0189680

**Published:** 2017-12-18

**Authors:** Penny Humaidah Hamid, Joko Prastowo, Ahmad Ghiffari, Anja Taubert, Carlos Hermosilla

**Affiliations:** 1 Department of Parasitology, Universitas Gadjah Mada, Karangmalang, Yogyakarta, Indonesia; 2 Faculty of Medicine, Universitas Muhammadiyah, Palembang, Indonesia; 3 Institute of Parasitology, Biomedical Research Centre Seltersberg, Justus Liebig University Giessen, Giessen, Germany; National Taiwan Ocean University, TAIWAN

## Abstract

*Aedes aegypti* is the primary vector of various relevant arthropod-borne viral infectious diseases worldwide. The mosquito control is still mainly performed by using insecticides but their effectiveness is increasingly questioned nowadays. We here conducted a study on *Ae*. *aegypti* resistance development towards several commonly used insecticides in the capital city of Jakarta, Indonesia. In order to achieve this goal, *Ae*. *aegypti* eggs from Jakarta were collected with ovitraps and hatched in the insectary of the Gadjah Mada University, Indonesia. The F0 generations were used for WHO resistance tests and knockdown resistance (kdr) assays. Presented results clearly showed that there was resistance development of *Ae*. *aegypti* populations to the here tested pyrethroid insecticides (i. e. permethrin). Observed mortalities were less than 90% with highest resistance against 0.75% permethrin concentrations. Furthermore, a significant association of V1016G gene mutations with resistance phenotypes to 0.75% permethrin was observed. Nevertheless, the F1534C mutation did not show a significant correlation to resistance development. In conclusion, our results show that populations of *Ae*. *aegypti* mosquitoes within the city of Jakarta have developed resistance against several routinely used pyrethroid insecticides in local performed control programs. Thus, the regular verification/assessment of resistance development status will hopefully help in the future to assist local public health authorities in their mosquito control programs by recommending and managing the rotation of different routinely used insecticides with diverse effector mechanisms in order to delay *Ae*. *aegypti* resistance development.

## Introduction

Dengue fever (DF) transmitted by the mosquito *Ae*. *aegypti* is still a major public health concern for human beings living in the Republic of Indonesia. So far, reported human DF cases have dramatically increased in the past decades in Indonesia. As such in 1968 only 58 human DF cases were reported to occur, whereas in the year 2015 a total of 126,675 DF cases were reported with a clear tendency of increased annual occurrence [1]. These increased new human DF cases in the last years show clearly that DF is still to be considered as a continuous threat to humans in Indonesia. Consequently, the scientific interest on *Ae*. *aegypti-*related investigations as main DF vectors, is nowadays rising in Indonesia also due to increased risks in transmitting also additional viral infectious diseases, i. e. Zika [2]. As reviewed elsewhere [3] the epidemiological spreading of Zika virus into previously non-endemic geographic regions and the rapid increase of reported human infections through continents is shocking and devastating. Besides increasing international travel activities of unapparent Zika-positive humans worldwide as an important risk factor [4–6], its spread within non-endemic countries is surely transmitted and secured by the presence of *Ae*. *aegypti* mosquitoes. *Ae*. *aegypti* mosquitoes are able to breed with ease also in subtropical countries which in the past were considered to be free of these arboviruses. Moreover, due to the acceleration of global warming, a significant increased of *Ae*. *aegypti* populations is expected to occur worldwide, thereby enhancing vectorial capacities which are unavoidably as postulated elsewhere [7]. Even the spread of non-infectious diseases, such as cancer, has also been reported to occur by mosquito bites of *Ae*. *aegypti* which mechanically transfered tumor cells from sick individuals into healthy ones *in vivo* [8].

Routinely performed vector control programs are still the most effective preventive means for arthropod-borne diseases since cures are expensive, mainly supportive and vaccines are still under experimental trials [9–11].

Controlling of *Ae*. *aegypti* populations in urban as well as suburban/rural areas by management strategies has been performed in the past by Indonesian government health authorities such as continuous campaigns for disposal of breeding sites. Currently, in many tropical countries larvitraps have been placed in airports for helping to reduce the risk of Zika introduction and its dissemination by National Health Agencies [12] even though the efficiency of this method is still under evaluation. Public awareness has sought to be improved through any level of education programs to encourage community participation since many years, however, incidence rates (IR) and DF outbreaks are annually regularly recorded since the beginning of the rainy season [13]. It has also been frequently shown that DF disease control through the abatement of *Ae*. *aegypti* populations, especially in geographic areas with tropical/subtropical climate conditions alongside the equatorial line supportive for this mosquito species, is still a worldwide challenge [14–18]. While performing national-planned mosquito elimination programs in Indonesia, mainly using pyrethroid-containing insecticides without any proper rotation schedules, the development of pyrethroid-derived resistance should be indeed expected to occur in permanently exposed vector populations in Indonesia.

Consistently, also in other tropical/subtropical countries insecticides are still used to combat mosquito vectors as the most feasible way since decades and therefore playing a key role in controlling mosquito-borne diseases such as malaria, DF, and human filariasis [19]. Insecticide-based vector controls have been challenged by the emergence of mosquito resistance to a variety of worldwide routinely used insecticides (e. g. pyrethroids of the first to fourth generation) reported worldwide not only for the order *Diptera* to which mosquitoes belong but also for other insect orders such as *Haemiptera*, *Phyraptera* and *Siphonaptera* with various modes of action [20]. Consistently, insecticide resistance development of various levels has been reported to occur also in urban/suburban insect populations with a clear anthropogenic behavior, living close and well-adapted to human environments [21]. These resistance issues become more problematic since only few new insecticides have been commercially developed because of high costs for their discoveries and further development [22]. Studies on *Ae*. *aegypti* resistance to insecticides are reportedly associated with knockdown resistance (kdr) mechanisms on the sodium voltage channel (Nav) of the mosquitoes [23], with at least seven point mutations, leading to reduced sensitivity of sodium voltage channels to routinely used insecticides [24].

Identification of *Ae*. *aegypti*-resistance related Nav gene shows that point mutations such as S989P, I1011M/V, V1016G/I, F1269C, F1534C correspond well to this resistance development in this mosquito species [23]. For instance, detailed regional analyses have shown that V1016I point mutations clearly contribute to pyrethroid resistance development in *Ae*. *aegypti* populations of South America [25,26]. In contrast to V1016I findings in South America, the V1016G mutation is more frequently to be found in populations of *Ae*. *aegypti* throughout Asia [15,16,27–30]. Additionally, synergistic resistance development may also occur by additional point mutations in different locations of the Nav gene as recently reported elsewhere [31].

Jakarta is the capital of Indonesia and as metropolitan city the most populous within the Indonesian Archipelago [32]. Nowadays, Jakarta is considered as top fifth endemic area with an average IR of 77.98 per 100,000 citizens in the years 2011–2013 [1]. The frequent usage of skin repellents and space-spraying insecticides among Jakarta citizens are still very high in order to avoid mosquito bites. Therefore, the periodically assessment of the resistance status of exposed *Ae*. *aegypti* populations in these urban areas is extremely important. Thus, we here investigated resistance development against diverse routinely used pyrethroids by characterizing present genotypes, distributions and variations of *Ae aegypti* Nav genes in Jakarta. These data will hopefully not only help to better understand resistance genetic development in *Ae*. *aegypti* in Indonesia, but also to serve as baseline survey for future monitoring studies on this relevant public health issue.

## Materials and methods

No ethical clearance was issued regarding these experiments since no blood feeding were provided and exclusively F0 mosquitoes were used for all experiments performed in this work.

### Mosquito samples

*Ae*. *aegypti* eggs were collected from Jakarta, the capital city of Indonesia. Ova were collected by using 50 artificial ovitraps placed in residential areas, i. e. Kebon Jeruk (6°11'46.4"S 106°45'52.5"E), Kebayoran Lama (6°15'06.9"S 106°47'05.4"E), Kebayoran Baru (6°15'40.2"S 106°48'05.7"E), Cempaka Putih (6°11'06.8"S 106°51'49.4"E), Kramat Jati (6°16'38.3"S 106°51'49.1"E), Makasar (6°16'13.7"S 106°52'30.1"E), Ciracas (6°21'01.0"S 106°52'13.3"E) and Cipayung (6°20'17.3"S 106°54'13.0"E).

The collection sites were randomly selected with particular emphasis on districts with previously reported human DF cases and regular fogging intensities. Water and filter papers of ovitraps were replaced every week during collection dates. Samples were collected from October to November 2016.

Egg-containing papers were dried and thereafter stored in plastic containers. Eggs were then immediately transported and hatched in the insectary of the Department of Parasitology, Veterinary Medicine, Gadjah Mada University, Indonesia. Hatched *Ae*. *aegypti*-larvae were fed with chicken liver (wet and dried) until reaching pupa and adult stages. Adult *Ae*. *aegypti* mosquitoes were fed with 10% sugar solution absorbed into cotton balls. Emerged F0 mosquitoes up to two days of age were used for all resistance-related experiments.

### Insecticide susceptibility test

Insecticide succeptibility test (IST) were performed according to WHO protocols for Anopheline mosquitoes diagnostic doses [33]. The kits and insecticide impregnated papers were prepared and supplied by the Vector Control Research Unit, Universiti Sains Malaysia as officially WHO collaborating centre in Asia. The insecticide impregnated papers consisted of 5% malathion, 0.05% deltamethrin, 0.75% permethrin, 0.05% λ-cyhalothrin, 0.1% bendiocarb and 0.15% cyflothrin, respectively. In each IST assay 150 alive mosquitoes from each city sites were divided into 6 tubes containing each 25 mosquitoes, respectively. Four tubes (4 replicates) served as replicates for 1 insecticide exposure and two tubes served as controls. The mortality is shown as percentage of the following formula: total number of dead mosquito/total sample size X 100 as previously described [33]. Abbott’s formula was not used in this study since control mortalities were always less than 5%. Some mosquitoes (dead and alive) from the assays were kept at -20°C for further molecular analysis. Resistance status of mosquito populations was defined according to the WHO recommended threshold of < 90% mortality [33].

### DNA isolation

Mosquito DNA isolations were performed individually by using the commercial DNeasy Blood and Tissue Kit^**®**^ (Qiagen, Germany) and performed according to manufacturer’s instructions. In addition, occasional vortexing using glass beads was performed during proteinase-K incubation (which is included in the kit) to ease mosquito lysis.

### V1016G and F1534C allele specific PCRs

Genotyping of the mutants V1016G and F1534C were performed according to previous publications [27,34] for allele specific PCR assays. For the V1016G detection the PCR consisted of 1 μl of 10 pmol forward primer 5’ACCGACAAATTGTTTCCC3’, 0.5 μl of 10 pmol of each reverse primer 5’GCGGGCAGGGCGGCGGGGGCGGGGCCAGCAAGGCTAAGAAAAGGTTAACTC3’ and 5’GCGGGCAGCAAGGCTAAGAAAAGGTTAATTA3’, 12.5 μl of Dream Taq Green PCR Master Mix^®^ (Thermo Fisher Scientific) in a 25 μl total reaction volume. Reactions were performed as follows: 94°C at 2 min, 35 cycles of 30 s at 94°C, 30 s at 55°C, 30 s at 72°C, and a final elongation step for 2 min at 72°C. PCR amplification products were then loaded onto a 3% agarose gel. The F1534C detection PCR consisted of 1 μl of 10 pmol forward primer 5’GCGGGCTCTACTTTGTGTTCTTCATCATATT3’, 0.5 μl of 10 pmol of forward primer 5’GCGGGCAGGGCGGCGGGGGCGGGGCCTCTACTTTGTGTTCTTCATCATGTG3’ and 1 μl of reverse primer 5’TCTGCTCGTTGAAGTTGTCGAT3’, 12.5 μl of Dream Taq Green PCR Master Mix^®^ (Thermo Fisher Scientific) in a 25 μl total reaction volume. Reactions were performed as follows: 94°C at 2 min, 35 cycles of 30 s at 94°C, 30 s at 60°C, 30 s at 72°C, and a final elongation step for 2 min at 72°C. PCR amplification products were then loaded onto a 3% agarose gel as above mentioned.

### Statistic analysis

Statistical analysis and graphical presentation were processed using software GraphPad Prism 7.02. *P* values of < 0.05 were considered as significant.

## Results

### Adult resistance to tested insecticides

**Fig 1:**
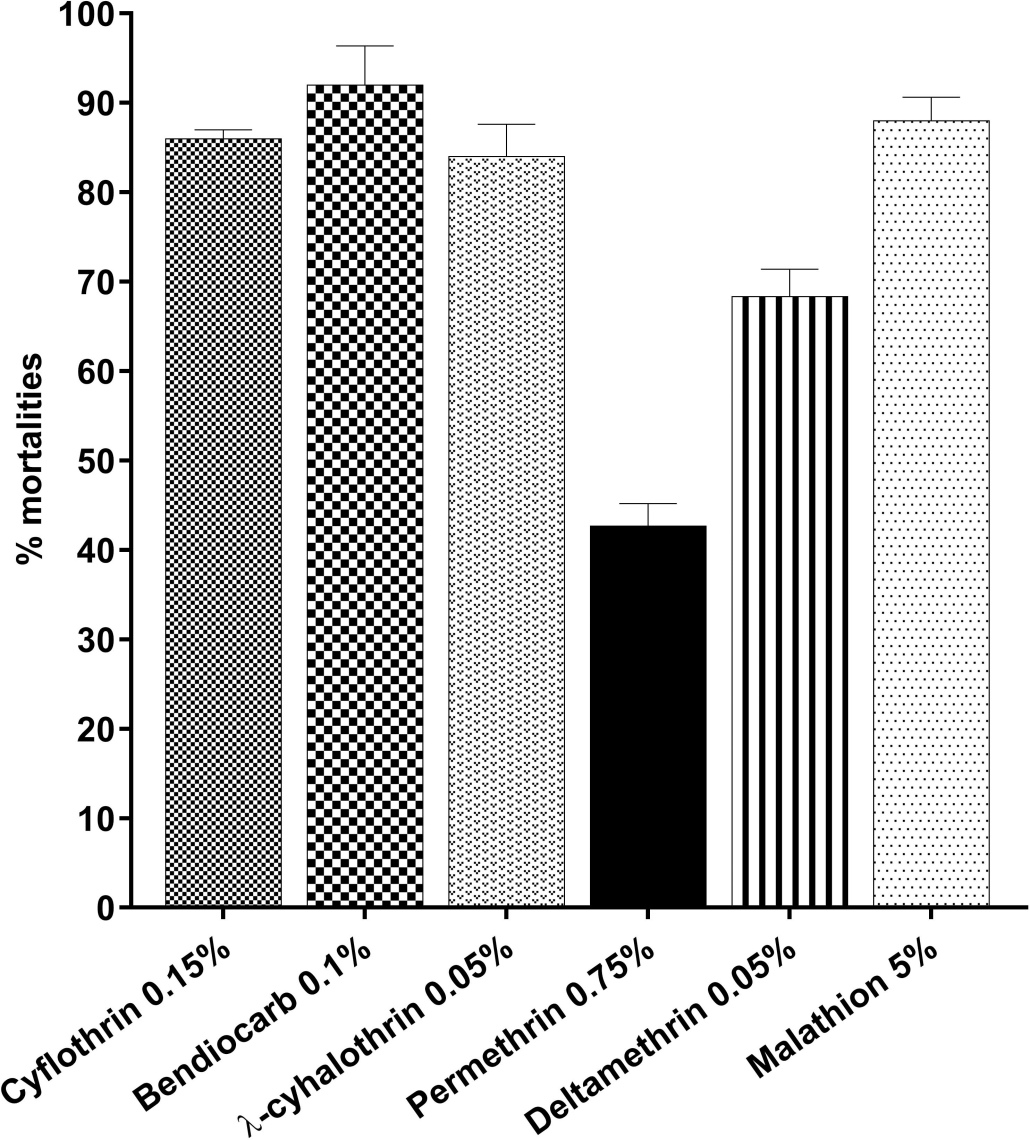
Resistance profiles of *Ae*. *aegypti* from Jakarta.

All tested mosquitoes from the different locations of Jakarta presented resistance levels to IST-tested insecticides as shown in Fig 1. Mosquito populations showed resistance to pyrethroid insecticides with mortalities less than 90% according to WHO protocols [33]. However the response to 0.1% bendiocarb was still above of 90% in tested mosquitos. There was a significant difference of the mosquito response to tested insecticides (*F*_(5,12)_ = 111.9, *P* < 0.0001). Highest resistance were observed against permethrin when compared to any other tested insecticides (0.05% deltamethrin *t* = 11.23, *P* < 0.001; 0.05% λ-cyhalothrin, *t* = 16.28, *P* < 0.0001; 5% malathion *t* = 21.5, *P* < 0.0001; 0.1% bendiocarb *t* = 16.28, *P* < 0.0001; 0.15% cyflothrin *t* = 27.72, *P* < 0.0001) and followed only by 0.05% deltamethrin. Mild *Ae*. *aegypti* resistance development was observed against 5% malathion, 0.05% λ-cyhalothrin and 0.15% cyflothrin.

### Mosquito genotyping

**Fig 2:**
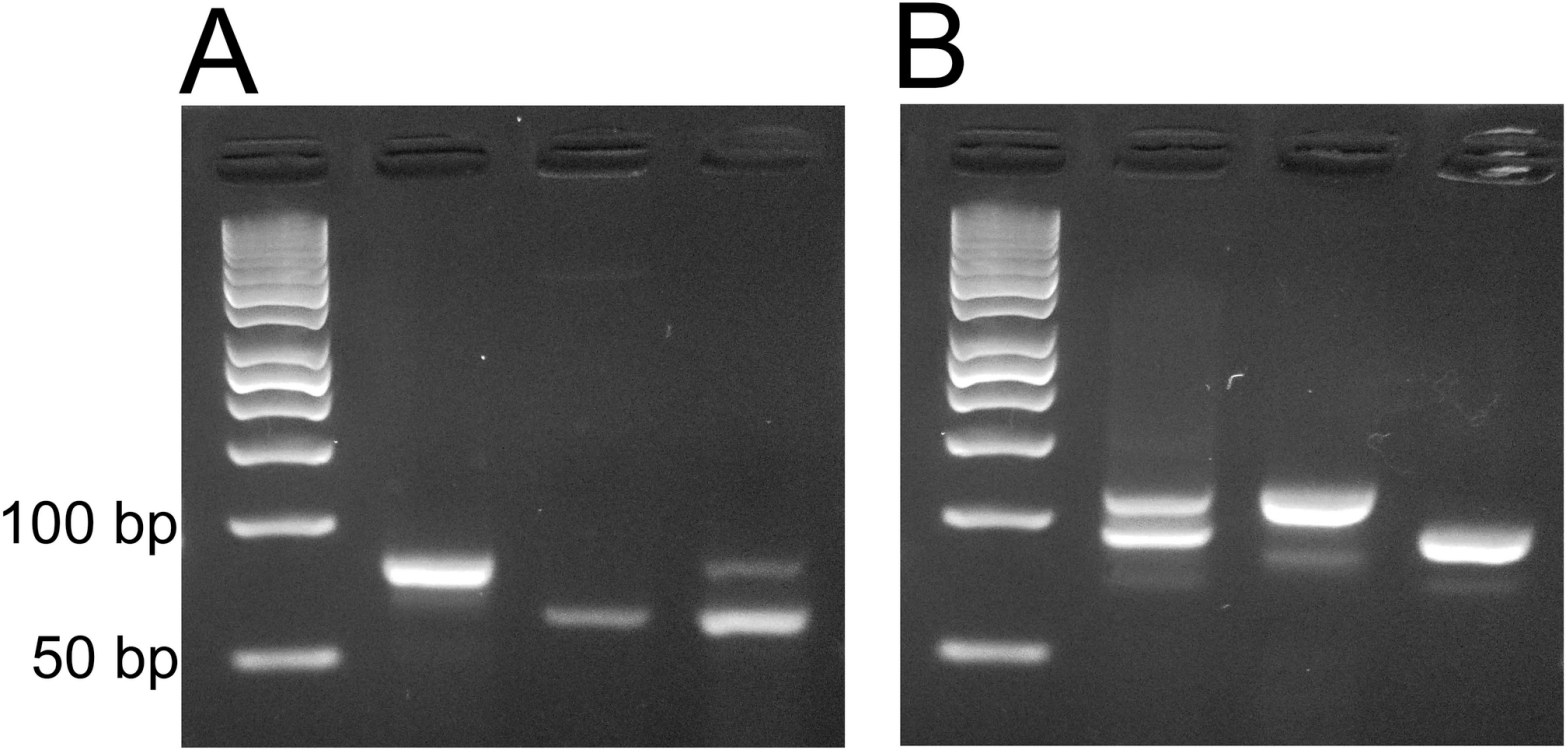
Allele-specific PCR exemplaries on agarose gel. (A) V1016G mutation, (B) F1534C mutation. Wt: Wild-type, Mh: Mutant homozygote, MHt: Mutant heterozygote, M: 50-bp DNA marker.

**Fig 3:**
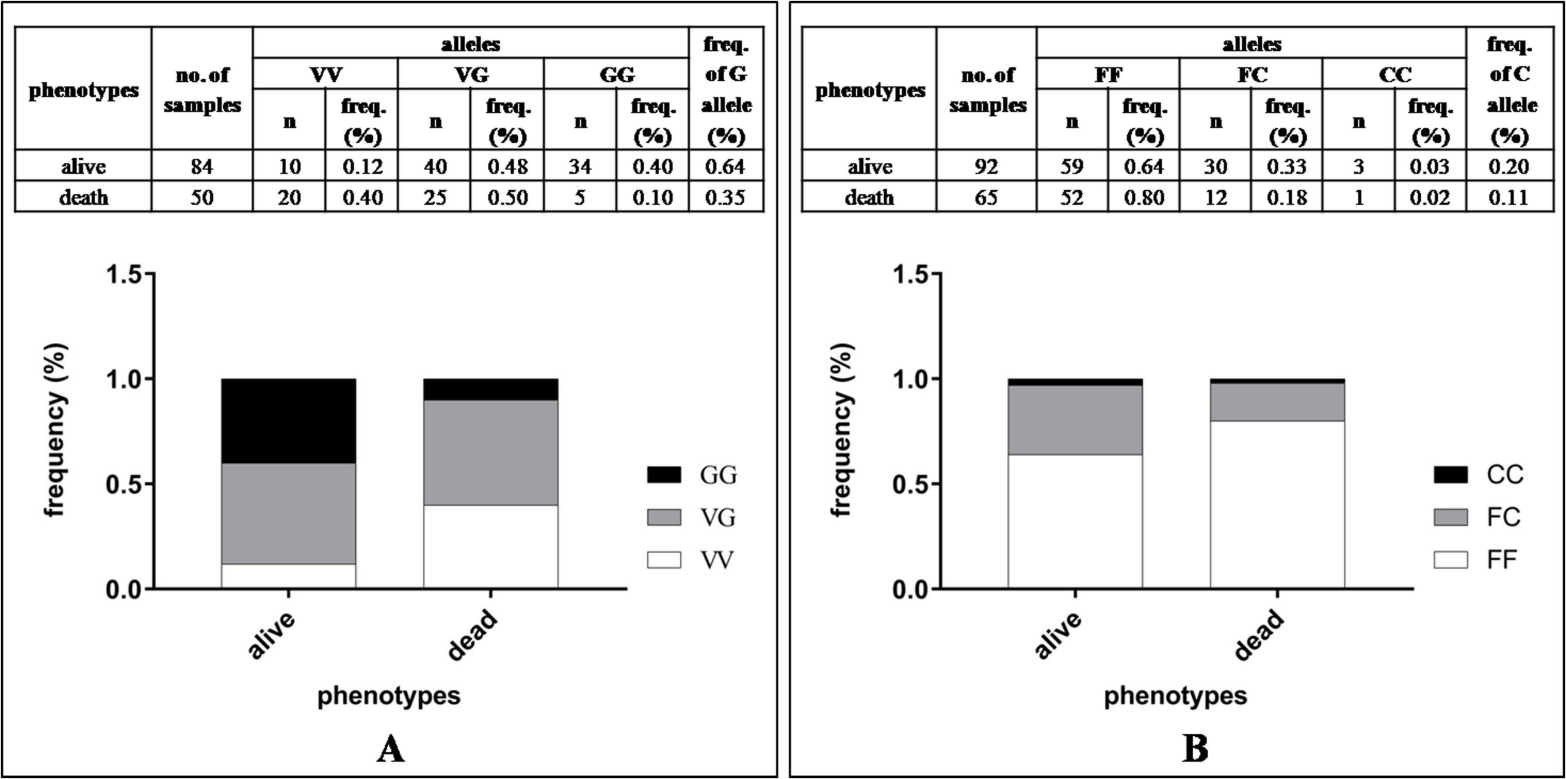
Phenotypes of *Ae*. *aegypti* population from Jakarta to 0.75% permethrin. (A). Ratio on V1016G frequency, (B). Ratio on F1534C frequency.

Allele specific PCRs showed clear results to describe mutant as well as wild-type expression of kdr genes in *Ae*. *aegypti* mosquitoes (please see Fig 2). Allele specific PCR of V1016G showed distributions of V and G point mutations in the Nav gene domain II. In the resistance (alive) phenotypes, the homozygotes frequencies of GG were 0.40% and the heterozygotes frequencies of VG were 0.48%, respectively. Conversely, susceptible (dead) mosquito samples showed GG frequencies of 0.10% and VG frequencies of 0.50% (see Fig 3A). Total frequency of G allele in the resistance phenotypes was 0.64% and 0.35% in the susceptible group of the *Ae*. *aegypti* population from Jakarta. The descriptive distribution pattern of G allele in the resistant and susceptible mosquitoes is provided in Fig 3A. Odd ratio (OR) of total V1016G mutations showed significant association (Fisher’s exact test, *P* = 0.0002, OR = 6.12, 95% CI = 2.31–15.29) with resistance phenotypes tested against permethrin (Table 1).

**Table 1:** Association of V1016G and F1534C with resistance to 0.75% permethrin.

type of mutation	phenotypes	genotype		OR (95% CI)	p value of Fisher's test
		**V1016V and V1016G**	**G1016G**		
V1016G	alive	50	34	6.12	0.0001 (S)
	dead	45	5		
		**F1534F and F1534C**	**C1534C**		
F1534C	alive	89	3	2.16	0.64 (NS)
	dead	64	1		

*(S) = significant p ≤ 0.05

**(NS) = not significant p ≤ 0.05

Allele specific PCR of F1534C showed distributions of F to C point mutations in the Nav gene domain III (see Fig 3B). In the resistant (alive) phenotypes, homozygotes frequencies of CC were 0.03% and in heterozygotes the FC frequencies were 0.033%. Susceptible (dead) samples showed CC frequencies of 0.02% and of 0.18% for FC frequencies (Fig 3.). Total frequency of C allele in the resistance phenotypes was 0.2% and 0.11% in the susceptible group of *Ae*. *aegypti* population from Jakarta. The descriptive distribution pattern of C allele in all mosquitoes is provided in Fig 3B. In contrast to GG allele, the CC allele was relatively rare detected in investigated *Ae*. *aegypti* population. Odd ratio (OR) of F1534C mutation showed no significant association (Fisher’s exact test, *P* = 0.64, OR = 2.16, 95% CI = 0.22–21.23) with phenotypes tested against permethrin (Table 1).

## Discussion

Controlling vectors is the only possible way to efficiently combat vector-borne diseases when adequate treatments and suitable vaccines for these diseases are not yet established. International as well national health authorities have been combating *Ae*. *aegypti* as major vectors of important viral diseases since many decades, nonetheless with limited success so far due to concomitant factors, among others the resistance development against insecticides. *Ae*. *aegypti* has passed evolutionary processes impacted by its relationship with human and evidently able to survive in various human-living environments [35]. This well human-adapted (anthropogenic) mosquito species spread initially from Africa to other continents extremely fast and nowadays known as one of the most aggressive invader (neozoa) species in new geographic areas in which it was introduced [36,37]. Additionally, even a re-invading scenario was evidenced in Brazil after massive national control programs which led to declare this ectoparasite as successfully eliminated from national territories [38]. Re-colonization of previously declared ‘mosquito-free geographic areas/nations’ is most probably occurring from areas in which mosquito eradication was never completely achieved [38] evidencing again that control programs have to be a nation/international teamwork effort and to be coordinated simultaneously between regions across country boundaries.

Consistently, the annual cyclic patterns of reported human DF cases in Indonesia [13] clearly demonstrate that this vector-borne disease is still difficult to combat. Cases occur in almost all parts of the provinces of the Indonesian archipelago, although some islands are separated geographically by wide straits and ocean in between. Current DF disease control failure is not exclusively reported to occur in Indonesia, but also to be described from other DF endemic countries with similar warm temperatures since climate is well-known to impact significantly on the vector abundance, the timing and the intensities of DF outbreaks as reviewed in detail elsewhere [39]. Nonetheless, vector control is undebatedly still considered as the key strategy to reduce IR of DF disease especially in geographic areas where humans act as the only transmission host. The use of chemical insecticides is the most powerful alternative chosen by many poor countries when there are still no other cheap options to prevent disease outbreaks so far. Therefore, the assessment of the resistance status of periodically insecticide-exposed mosquitoes is urgently needed and to be performed regularly in order to choose appropriate insecticides in the local operational acts.

In this study, the V1016G point mutation was distributed in a rather high frequency, both in resistance (alive) and susceptible (dead) mosquitoes. This haplotype is commonly found in *Ae*. *aegypti* from Asia [15,16,27–29] but until now still not found in South American *Ae*. *aegypti* populations, where the point mutation 1016Ile is the most dominant one [25]. In this study F1534C was less frequently found in comparison to V1016G and which is not associated with resistance pattern to permethrin. This is in line with previous reports in Java [15,16] and our recent report in Bali [14], but in contrast with recently published data, where the F1534 C point mutation also significantly contributed to the type I phyrethroid resistance development in *Ae*. *aegypti* mosquitoes [40]. In this context, the presence of resistant phenotypes in the wildtype genotypes may due to the metabolic resistance mechanisms known to occur in mosquito populations [41]. Worthy of mention is the fact that dual point of mutations in mosquitoes are known to have not only a significant effect on resistance development but also can severely enhance the strength of insecticide resistance as reported elsewhere [42].

Undoubtedly, at least two pyrethroids used in this IST-related study were no more appropriate/effective against *Ae*. *aegypti* and thus not recommended to be utilized as routinely insecticides in future mosquito control campaigns within the city of Jakarta. More importantly, this study adds new data on the current resistance status of *Aedes* mosquitoes, which have a global distribution alongside the equatorial line [43], but unfortunately nowadays spreading into previously non-endemic areas as previously reviewed [44]. Taking into account that only few efficient insecticides against *Ae*. *aegypti* are yet available and that resistance development is increasing worldwide, unambiguously this should result in the careful use and rotation and/or combination of efficient drugs in order to prolong their current efficacy. A wise regulation in using insecticides is mandatory since resistance expression may arise after an intensive exposure [18]. Besides, alternate bio-insecticides may be promising in the near future as eco-friendly repellent with minimum negative effects for humans and environment. Whilst no new insecticides are released into the market, the novel approach to accelerate the delivery of certain insecticides to their specific target sites within the mosquito, i. e. by utilizing various nanoparticles as carriers, seems a promising approach [45]. In addition, the elimination of adequate mosquito breeding sites in urban/suburban areas, as compulsory performed by some Asian city authorities (e. g. Singapore) are urgently needed and should to be managed properly as an integrative work of local as well as national health authorities. For instance, in the city of Jakarta where wetlands are regularly increased in sizes during the rainy seasons and coupled with limited catchment area, will obviously facilitate dramatically the breeding and propagation of urban *Ae*. *aegypti* populations. Development and propagations of sterile insect technique may enhance ability to control insect populations in a limited (narrow) area [46]. Gene–edited mosquitoes may be becoming easier to be produced with the now being well established CRISPR/Cas method [47]. Surely, the release of gene-edited organisms needs further long consideration and evaluation on their effects on a population level. Biological control efforts with *Wollbachia*-enginered mosquitoes, which have already been released in the city of Yogyakarta, Indonesia, [15] is a promising alternative to insecticide-based mosquito control but needs further evaluation.

## Conclusions

The here investigated *Ae*. *aegypti* population of Jakarta is clearly resistant to several frequently used insecticides and with mortalities less than 90%. As *Ae*. *aegypti* is still considered as the main vector of several arthropod-borne viral infections in the tropics/subtropics profoundly affecting humans the assessment of detected insecticide resistance should be tested more regularly by national public health authorities in Indonesia. Consequently, we here call for more investigations on this important entomological field as insecticide resistance could clearly spread rapidly into other urban areas of the most populated island of the Indonesian archipelago, namely Java. Furthermore, we strongly recommend assessments and verification on the resistance status of *Ae*. *aegypti* populations not exclusively against pyrethroids but also other insecticides with different effector mechanisms frequently used which will hopefully help to plan and manage in the future the appropriate use of efficient insecticides in local and national mosquito control campaigns.
